# School-level economic disadvantage and obesity in middle school children in central Texas, USA: a cross-sectional study

**DOI:** 10.1186/1479-5868-12-S1-S8

**Published:** 2015-07-27

**Authors:** Andrew E Springer, Linlin Li, Nalini Ranjit, Joanne Delk, Kajal Mehta, Steven H Kelder

**Affiliations:** 1 Michael & Susan Dell Center for Advancement of Healthy Living, University of Texas School of Public Health-Austin Regional Campus, TX, USA; 2 Texas Department of State Health Services, Austin, TX, 78756, USA; 3 UT Southwestern Medical Center, Dallas, TX, USA

## Abstract

**Background:**

Although children of lower socio-economic status (SES) in the United States have generally been found to be at greater risk for obesity, the SES-obesity association varies when stratified by racial/ethnic groups-with no consistent association found for African American and Hispanic children. Research on contextual and setting-related factors may provide further insights into ethnic and SES disparities in obesity. We examined whether obesity levels among central Texas 8^th^ grade students (n=2682) vary by school-level economic disadvantage across individual-level family SES and racial/ethnicity groups. As a secondary aim, we compared the association of school-level economic disadvantage and obesity by language spoken with parents (English or Spanish) among Hispanic students.

**Methods:**

Multilevel regression models stratified by family SES and ethnicity were run using cross-sectional baseline data from five school districts participating in the Central Texas CATCH Middle School project. For *family SES*, independent multi-level logistic regression models were run for total sample and by gender for each family SES stratum (poor/near poor/just getting by, living comfortably, and very well off), adjusting for age, ethnicity, and gender. Similarly, multi-level regression models were run by *race/ethnic group* (African American, Hispanic, and White), adjusting for age, family SES, and gender.

**Results:**

Students attending highly economically disadvantaged (ED) schools were between 1.7 (95% CI: 1.1-2.6) and 2.4 (95% CI: 1.2-4.8) times more likely to be obese as students attending low ED schools across family SES groups (p<.05). African American (OR_Adj_ =3.4, 95% CI: 1.1-11.4), Hispanic (OR_Adj_=1.8, 95% CI 1.1-3.0) and White (OR_Adj_=3.8, 95% CI: 1.6-8.9) students attending high ED schools were more likely to be obese as counterparts at low ED schools (p<.05). Gender-stratified findings were similar to findings for total sample, although fewer results reached significance. While no obesity differences across school ED categories were found for Hispanic Spanish-speaking students, Hispanic English-speaking students (HES) attending high ED schools were 2.4 times more likely to be obese as HES students at low ED schools (p=.003).

**Conclusion:**

Findings support the need to prioritize economically disadvantaged schools for obesity prevention efforts and support further exploration of school SES context in shaping children’s physical activity and dietary behaviors.

## Background

Despite initial evidence of a leveling off of childhood obesity in the United States [1], ethnic and socioeconomic inequalities in childhood obesity have persisted. Between 2001 and 2010, African American and Hispanic children and children of lower socioeconomic status (SES) were consistently found to have a significantly higher prevalence of childhood obesity compared to White American and higher SES children, respectively [2]. The persistence of ethnic and SES disparities in childhood obesity in the United States is supported by a large body of research that documents the greater burden of childhood obesity in African American and Hispanic children [1,3,4] as well as children of lower SES [3,5,6].

Although children and adolescents of lower SES have generally been found to be at greater risk for childhood obesity, the association of obesity and SES has been found to vary when stratified by racial/ethnic and gender groups. Findings based on the National Health and Nutrition Examination Survey (NHANES) between 1971 and 2002 indicate that family SES is inversely related to obesity in White children aged 2-18 years, but not among African American or Mexican American children [6]. Important gender-specific inequalities in the SES-obesity association across ethnic groups were also noted [6]. Analysis of the 2003 National Survey of Children’s Health data provides further evidence of ethnic and gender differences in the SES-obesity association in U.S. children and adolescents. Although adjustment for family SES eliminated ethnic differences in obesity in comparing Hispanic girls aged 10-17 years with same-aged white girls, Hispanic boys as well as African American adolescents maintained a higher risk for obesity after SES adjustment [7]. These mixed findings on the family-level SES-obesity association when stratified by ethnicity and gender are supported by other studies of U.S. children and adolescents [8,9] and underscore the complex nature of the obesity-SES association in U.S. children and adolescents.

Research on contextual and setting-related factors may provide further insights into ethnic and SES inequalities in childhood obesity as well as the inconsistency in findings by race/ethnicity and gender. Ecological models of health posit that health and behavior are shaped by the interaction between the individual and his/her environment, defined broadly as the space “outside the individual” [10]. This space includes various types of environments (e.g., social, policy, built and information environments) as well as the settings (e.g., home, school, and neighborhood) that encompass those environments. An emerging evidence base suggests that settings may directly influence obesity and obesity-related behaviors. Findings from the *Moving to Opportunity* study-an experimental study in which low-income families in five U.S. cities were randomized to live in high or low poverty neighborhoods, for example, found that moving to a low poverty neighborhoods was associated with reduction in extreme obesity and diabetes among low income women [11]. Further research is needed to understand how children’s environments and settings shape obesity and related behaviors as well as how the impact of these contexts may differ by individual factors such as family SES and race/ethnicity.

Schools represent a key developmental context and setting for children and adolescents that hold potential to influence children’s obesity-related behaviors [12]. Although research is limited on school-level SES effects among adolescents by race/ethnicity, an emerging evidence base suggests that the school a child attends may influence his or her energy-balance behaviors as well as obesity. Richmond, et al. [13], using nationally representative data on U.S. high school students, found that ethnic differences in adolescents’ physical activity engagement were a function of where adolescents attended school. While White girls and boys were generally found to engage in more physical activity, White girls engaged in similar levels of physical activity as their Hispanic and African American counterparts when attending lower income schools where Hispanics and African American were the majority. White boys in the same lower income schools, on the other hand, participated in less physical activity than African American and Hispanic boys [13]. Other recent cross-sectional evidence from the U.S. indicates that elementary school children in Oregon [14] and middle school girls in Minnesota [15] who attend lower SES schools have a significantly higher prevalence of obesity compared to students who attend higher SES schools. Further research is needed to understand the role of school-level SES inequalities in shaping childhood obesity risk for individuals of low SES as well as individuals from ethnic groups that are at higher risk for obesity.

This study examines whether obesity levels among central Texas middle school students from diverse family SES and ethnic backgrounds vary by school-level SES, as measured by the proportion of economically disadvantaged students at a given school. The specific study questions we aimed to address were: *Do low income adolescents attending less economically disadvantaged schools (low ED) have lower obesity prevalence compared to low income students attending more economically disadvantaged schools (high ED)? Do specific ethnic groups of adolescents (African American, Hispanic and White) attending low ED schools have lower obesity prevalence compared to same-ethnic group students attending high ED schools?* As a secondary aim, we also compared the association of school-level economic disadvantage and obesity by language spoken with parents (English or Spanish) among Hispanic students.

## Methods

### Data source and study population

A secondary analysis was conducted using cross-sectional baseline data collected in spring 2009 from the Central Texas CATCH Middle School Project (“CATCH Middle School”), a three and a half year coordinated school-based health promotion project aimed at promoting physical activity and healthy eating in middle school students from five school districts in central Texas [16]. Thirty middle schools selected from a universe of 32 schools located in five central Texas independent school districts comprised the study sample of schools. In spring 2009, a total of n=2,826 8^th^ grade students participated in the baseline study. Students were recruited via verbal and written invitations to participate in the study through core classes that all students in a given school must attend, such as advisory period, science, math or English classes. Student assent and parental consent were obtained for all students. The original study was reviewed and approved by the University of Texas Health Science Center at Houston Committee for the Protection of Human Subjects and participating school district internal review boards and is in accordance with the ethical standards of the Helsinki Declaration.

### Measures and study variables

#### BMI classification

Trained and certified research staff measured height with a portable stadiometer and weight with a portable digital scale following standard protocols that have been reported previously [16,17]. Body Mass Index (BMI) classification, the primary dependent variable, was calculated using the CDC growth charts that take into account children’s age and sex [18]. Based on the CDC definition for obesity, we classified children as *obese* (≥ 95^th^ percentile) and *non-obese* (<95^th^ percentile).

#### School-level economic disadvantage

*School-level economic disadvantage* (“School ED”), the primary independent variable, was based on the percentage of a given school’s student population that were economically disadvantaged. The economically disadvantaged classification is defined by the Texas Education Agency (TEA), based on National School Lunch and Child Nutrition Program eligibility for free or reduced-priced meals, as families with incomes at or below 130% or between 130% and 185% of the federal poverty level, respectively [19]. Economic disadvantage scores were obtained from the TEA for the 2009-2010 school year, and all students attending a given school were assigned that school’s overall economic disadvantage score. ED scores in schools ranged from 10.4% to 95.8%. Three categories were created based on the tertile distribution of economic disadvantage for the overall sample: low school-level ED (10.4-41.7% composition of economically disadvantaged students), medium school-level ED (>41.7-≤74.2%) and high school-level ED (>74.2%). Each category included 10 schools.

#### Student socio-demographic characteristics

Students completed a self-administered questionnaire that included closed-ended items on energy balance related behaviors and socio-demographic characteristics (see Springer, et al., [16] for details). This study focuses specifically on socio-demographic measures related to *individual-level SES* (“family SES”), *race/ethnicity, gender and age*.

*Family SES* was based on a single item measure in which students rated their family’s economic standard of living. This measure has been found to have evidence of construct validity based on studies of youth risk behavior in the U.S. and El Salvador [20-22]. Students were asked: “In terms of income, what best describes your family’s standard of living in the home where you live most of the time?” Response options included: poor, nearly poor, just getting by, living comfortably, and very well off. Due to small cell size in the “poor” and “nearly poor” categories, we combined these categories with “just getting by”, resulting in three categories: “poor-just getting by”, “living comfortably” and “very well off.”

##### Race/Ethnicity

Students were asked to describe themselves by choosing from a list of ten possible response options. For this study, we focused on the three largest ethnic groups: Black or African American (“African American”); Mexican-American, Latino or Hispanic (“Hispanic”); and White, Caucasian or Anglo (“White”). “Other” racial/ethnic group students were included in family SES analyses with the aim of generalizing back to the central Texas middle school population.

*Other Socio-Demographic Characteristics* assessed included age, measured by asking students to fill in their birth date, gender (male/female), and language spoken with parents most of the time (English/Spanish).

### Statistical analysis

Descriptive statistics were based on percentages for categorical outcomes, and means and standard deviations for continuous outcomes. Multi-level logistic regression analyses, accounting for school-level clustering [23], were conducted to assess differences in the prevalence of obesity by socio-demographic characteristics and by school economic disadvantage. As a measure of association between a given factor and obesity prevalence, unadjusted Odds Ratios (OR) were computed for the socio-demographic analyses, and adjusted Odds Ratios (AOR) were computed for associations between school economic disadvantage and obesity, adjusting for factors described below. In assessing the association of school economic disadvantaged and obesity by family SES and race/ethnicity, two separate sets of analyses were conducted. For *family SES*, independent multi-level logistic regression models were run for the total sample and stratified by gender to examine the association of school-level economic disadvantage and obesity within each family SES category (poor/near poor/just getting by, living comfortably, and very well off), adjusting for age, race/ethnicity, the interaction between family SES and school SES, and gender (total sample only). Similarly, independent multi-level regression models were run by *racial*/*ethnic group* (African American, Hispanic, and White) for the total sample and stratified by gender, adjusting for age, family SES, the interaction of school ED and family SES, and gender (total sample only). Statistical analyses were conducted with SAS software (SAS Institute Inc., Version 9.2, Cary, NC), with statistical significance set at a p-value of <.05.

## Results

The final analytic sample was n=2,682 students, after excluding missing values for the principal outcome variable. Students had a mean age of 13.9 years (SD: ±0.6), and just under half (48.8%) were female (Table 1). A higher percentage of boys (21.5%) were obese compared to girls (16.5%) (p<.001). The ethnic composition of students was diverse and included Hispanic (52.0%), White (25.1%), African American (13.1%), and “Other” (9.8%) ethnic groups. Hispanic (24.6%) and African American (18.9%) children had the highest prevalence of obesity, followed by White (12.5%) and “other” (8.8%) ethnic groups. A higher proportion of students attending lower economically disadvantaged (ED) schools (i.e., economically better-off schools) reported being “well off” (20.8%) compared to students attending medium ED (13.9%) and high ED (13.5%) schools [data not shown]. While no significant differences in prevalence of obesity were observed by family SES in unadjusted analyses, students attending medium and higher ED schools were significantly more likely to be obese compared to students attending lower ED schools, with similar findings by gender, in the unadjusted analyses (Table 1).

**Table 1:** Socio-demographic characteristics and obesity prevalence of 8th grade student sample (n = 2682). *Central Texas CATCH Middle School Project*, Spring 2009.

	Sample		% Obese		
	(n = 30 schools)		Total		
	n	%	%	Crude OR (95% CI)	p-value
n of students	2682	100%	19.20	--	--
Age in years (mean ± SD)	2679	13.88±0.60	13.86±0.63	--	--
Gender					
Female (ref.)	1308	48.77	16.47	1.00	--
Male	1374	51.23	21.51	1.39(1.14, 1.69)	0.001
Ethnicity					
White (ref.)	663	25.05	11.37	1.00	--
African American	347	13.11	21.05	2.10(1.46,3.01)	<0.0001
Hispanic	1377	52.02	22.87	2.31(1.74,3.07)	<0.0001
Other	260	9.82	15.07	1.40(0.92,2.12)	0.12
Speaks Spanish with parents most of time	764	29.02	20.57	1.15(0.92,1.44)	0.23
Family SES					
Very Well Off (ref.)	390	15.94	18.23	1.00	--
Living Comfortably	1510	61.73	18.34	1.01(0.75,1.35)	0.96
Poor/Just Getting By	546	22.32	18.10	0.99(0.71,1.40)	0.96
School Econ. Disadv.					
Low School ED (ref.)	872	32.51	12.27	1.00	--
Medium School ED	912	34	20.83	1.88(1.45,2.44)	<0.0001
High School ED	898	33.48	24.28	2.29(1.77,2.97)	<0.0001

Abbreviations: SES, socioeconomic status; School Econ. Disadv., School-Level Economic Disadvantage; ref., referent; OR, Odds Ratio.

Obesity defined as ≥95th percentile; family SES based on student self-report; school-level economic disadvantage based on Texas Education Agency data and on tertile distribution.

### Obesity, school economic disadvantage and *family SES*

**Figure 1:**
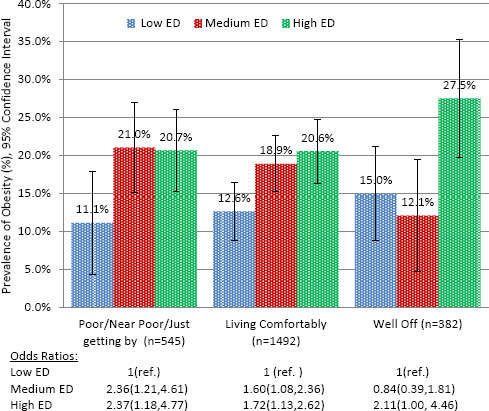
Prevalence and adjusted Odd Ratios* of obesity by school economic disadvantage (ED) stratified by family SES (Total, n=2,682). The Central Texas CATCH Middle School Project, Spring 2009. *Adjusted for age, gender, ethnicity.

Across family SES groups, students attending economically better-off schools (“low ED”) had significantly lower obesity prevalence compared to students attending the high ED schools (p<.05) (Fig. 1). The largest disparity in obesity was found for students who self-described as “poor - just getting by”, with poorer students attending high economically disadvantaged schools 2.4 times as likely to be obese as poor students attending low ED schools (95% Confidence Interval [CI]: 1.18, 4.77).

Patterns of the distribution of obesity by school ED across family SES groups were similar in gender-stratified analyses, although fewer associations reached statistical significance (Table 2). The largest difference in obesity prevalence across school ED was found for girls who self-described as “well off”. Well-off girls attending high ED schools were 5.7 times (95% CI: 1.43, 22.77) as likely to be obese as well-off girls attending low ED schools. No significant interaction effects were found for the total sample or gender stratified analyses based on the inclusion of an interaction term of family SES and school ED [data not shown].

**Table 2:** Prevalence of obesity by school-level economic disadvantage (ED) among central Texas 8th grade students, stratified by gender and family SES. *Central Texas CATCH Middle School Project*, Spring 2009 (n=2,644 students, n=30 public middle schools)

	**Girls**											
	*Poor - Just Getting By*				*Living Comfortably*				*Well Off*			
	n	% Obese	AOR (95% CI)	p-value	n	% Obese	AOR (95% CI)	p-value	n	% Obese	AOR (95% CI)	p-value
Low School ED (ref.)	71	8.78	1.00		274	10.49	1.00		86	8.97	1.00	
Medium School ED	93	19.67	2.60 (0.94,7.22)	0.09	250	14.90	1.49 (0.78,2.86)	0.28	52	18.02	2.40 (0.67,8.59)	0.22
High School ED	128	19.98	2.62 (0.91,7.56)	0.10	258	17.38	1.72 (0.88,3.37)	0.11	49	31.26	5.70 (1.43,22.77)	0.01
	**Boys**											
	*Poor - Just Getting By*				*Living Comfortably*				*Well Off*			
	n	% Obese	AOR (95% CI)	p-value	n	% Obese	AOR (95% CI)	p-value	n	% Obese	AOR (95% CI)	p-value
Low School ED (ref.)	70	11.87	1.00		239	15.22	1.00		84	20.72	1.00	
Medium School ED	87	22.64	2.20 (0.89,5.48)	0.10	267	23.23	1.70 (1.01,2.84)	0.05	57	11.15	0.45 (0.15,1.33)	0.18
High School ED	96	22.64	2.18 (0.85,5.60)	0.12	204	22.88	1.65 (0.93,2.94)	0.11	54	27.73	1.38 (0.53,3.59)	0.37

Abbreviations: ED, School-Level Economic Disadvantage; SES, Family Socioeconomic Status; ref., referent; AOR, Adjusted Odds Ratio; 95% CI, Confidence Interval. Obesity defined as ≥95th percentile; school-level ED based on Texas Education Agency data; family SES based on student self-report. Analyses adjusted for age and ethnicity.

### Obesity, school economic disadvantage and *race/ethnicity*

**Figure 2:**
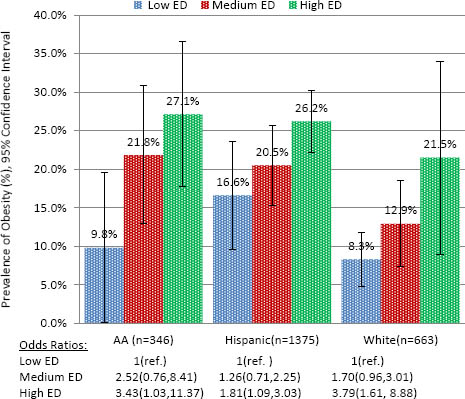
Prevalence and adjusted Odds Ratios* of obesity by school economic disadvantage (ED) stratified by ethnicity (Total, n= 2,682). The Central Texas CATCH Middle School Project, Spring 2009. *Adjusted for age, gender, family SES.

The prevalence of obesity was significantly higher among students attending higher economically disadvantaged schools across White, African American, and Hispanic racial/ethnic groups compared with same-ethnic group students attending low ED schools (p<.05) (Fig. 2). White and African American students attending high ED schools were approximately 3.7 times more likely to be obese compared to same-ethnic group students attending low ED schools (p<.05). Hispanic students attending high ED schools were also significantly more likely to be obese compared to students in low ED schools, although the magnitude of the association was lower (AOR: 1.57, 95% CI: 1.04, 2.37). In exploring findings for Hispanic students by Spanish/English language use, we found no significant differences in obesity prevalence across school ED categories among students who spoke Spanish with parents (23.3% for students attending medium and high ED schools; 24.5% for students attending low ED schools). Hispanic students who spoke English with parents and attended high ED schools, on the other hand, were 2.4 times more likely to be obese compared to Hispanic students who spoke English with parents and attended low ED schools (95% CI: 1.35, 4.31; p=.003) (obesity prevalence: 28.8% high ED, 22.1% medium ED, 14.2% low ED schools) [data not shown in tables].

In gender stratified analyses, patterns of obesity generally followed the same patterns for the total sample, with the prevalence of obesity tending to be higher in girls and boys in the high ED schools (Table 3). As with the gender-stratified family SES analyses, fewer differences reached statistical significance. African American and White girls who attended high ED schools were 4.9 and 8.7 times, respectively, as likely to be obese compared with same-ethnic group girls who attended low ED schools (p<.01). While obesity prevalence among Hispanic girls was higher in medium and high ED schools, findings were not statistically significant. No significant differences in obesity among boys across the three ethnic groups were noted. With the exception of white students in the total sample, no significant interaction effects were found based on the inclusion of an interaction term of family SES and school ED [data not shown]. For white students, the impact of school ED on obesity was found to be influenced by the level of their family SES (p<.02), while this combined effect was not present among AA and Hispanic students.

**Table 3:** Prevalence of obesity by school-level economic disadvantage (ED) among central Texas 8th grade students, stratified by gender and ethnicity. *Central Texas CATCH Middle School Project*, Spring 2009 (n=2,384 students, n=30 public middle schools)

	**Girls**											
	*African American*				*Hispanic*				*White*			
	n	% Obese	AOR (95% CI)	p-value	n	% Obese	AOR (95% CI)	p-value	n	% Obese	AOR (95% CI)	p-value
Low School ED (ref.)	34	9.40	1.00		110	14.04	1.00		236	6.00	1.00	
Medium School ED	68	17.91	2.10 (0.53,8.34)	0.34	245	20.06	1.53 (0.73,3.17)	0.27	80	13.64	2.44 (1.00,5.97)	0.055
High School ED	50	33.87	4.86 (1.21,19.50)	0.01	383	20.60	1.56 (0.76,3.19)	0.21	26	34.97	8.69 (2.98,25.33)	<.0001
	**Boys**											
	*African American*				*Hispanic*				*White*			
	n	% Obese	AOR (95% CI)	p-value	n	% Obese	AOR (95% CI)	p-value	n	% Obese	AOR (95% CI)	p-value
Low School ED (ref.)	34	7.69	1.00		102	21.24	1.00		208	20.72		
Medium School ED	86	23.01	3.24(0.73, 14.27)	0.10	228	24.39	1.19 (0.64,2.23)	0.59	99	11.15	1.33 (0.63,2.80)	0.47
High School ED	74	20.41	2.95(0.64, 13.53)	0.19	307	28.82	1.50 (0.82,2.73)	0.18	14	27.73	0.77 (0.09,6.44)	0.77

Abbreviations: ED, School-Level Economic Disadvantage; SES, Family Socioeconomic Status; ref., referent; AOR, Adjusted Odds Ratio; 95% CI, Confidence Interval. Obesity defined as ≥95th percentile; school-level ED based on Texas Education Agency data; family SES based on student self-report. Analyses adjusted for age and family SES.

## Discussion

This study examined the association of school-level economic disadvantage and obesity in middle school students in central Texas in the United States. A unique contribution of this study was the exploration of school-level economic disadvantage and obesity by adolescents’ individual-level SES and race/ethnicity based on a large sample of economically and ethnically diverse children from five school districts and 30 middle schools in central Texas. We found that students who attended schools that serve a greater proportion of economically disadvantaged students had higher obesity prevalence, regardless of their individual (family-level) economic status and/or ethnic background. These findings mirror prior research on the inverse association of school-level SES and obesity in elementary school children in Oregon [14] and middle school girls in Minnesota [15] and provide a foundation for prioritizing lower SES schools for obesity prevention efforts in the United States.

In exploring the mechanisms for the association between school-level SES and obesity, we cannot completely rule out that the associations observed resulted from a compositional effect, in which the effects of school-level economic disadvantage result directly from family-level SES of children who attend the schools. Although we adjusted for family-level SES in our analyses, our single item measure may fail to fully capture the various dimensions of family SES. In addition, we found that the association between school ED and obesity was influenced by the level of family SES among white students. In support of the importance of family level SES for understanding childhood obesity, several studies based on nationally representative time trend data from the United States have found that children from lower SES households engage in dietary practices that may contribute to higher obesity, which include higher consumption of energy-dense foods such as pizza, energy dense snacks, and higher consumption of sugar-sweetened beverage [24-26]. Food-related parenting practices may explain in part SES differences in obesity, with some research indicating that food-related parenting practices differ by SES [27,28]. Other factors that merit further exploration for understanding SES differences in child eating practices include infrequent family meals-given evidence of an inverse association of family meals with diet quality and obesity [29,30], and “time poverty”, given recent research that indicates that maternal employment is associated with less time spent grocery shopping, cooking, eating and playing with children [31]. An important finding reported by Ranjit and colleagues [32] in this journal supplement is that home food environment practices, assessed by availability of healthy foods at home, consumption of family meals, not watching television, not watching during meals, and lower frequency of eating at restaurants, may reduce SES disparities in children’s diets, particularly unhealthy food consumption.

While we cannot discard a compositional explanation for the obesity differences observed, merely increasing family SES may not solve the obesity issue. Gordon-Larsen and colleagues [33] estimated the effects of increasing family income and parental education on reducing racial/ethnic disparities in obesity based on nationally representative data of U.S. adolescents. They found only limited effects on reduction in racial/ethnic disparities of obesity and concluded that efforts must look beyond individual-level SES to other factors that include environmental and contextual factors. In support of contextual SES effects on child outcomes, some research has found that poor children who live in higher SES neighborhoods have better educational outcomes and fewer problem behaviors than poor children who live in lower SES neighborhoods [11,34,35]. Our findings of lower obesity prevalence among low income children and African American and Hispanic children who attend economically better off schools provide further foundation for the exploration of contextual factors, specifically SES contextual factors, for understanding childhood obesity.

As children in the U.S. spend the majority of their day and year in school [36], the school setting represents a key context that holds potential to establish social norms and shape child health behaviors. In examining differences in health-related outcomes by race/ethnicity among fifth grade students in three metropolitan areas in the U.S., Schuster and colleagues [37] found that adjustment for the school the child attends-in addition to household income and household education level-substantially reduced if not eliminated racial/ethnic disparities in health outcomes, including obesity. Our findings contribute to a small but growing body of literature from the United States on disparities in obesity and energy-balance related behaviors by school SES [13-15,37] and underscore the potential role schools play in fostering or preventing childhood obesity.

Ecological models of health behaviors [10] provide a framework for exploring school-setting effects on obesity and obesity-related behaviors. At the *school interpersonal level*, factors such as social comparison and social influence may shape individual weight perceptions and obesity-related behaviors [29,38]. It is possible that students and parents are both influenced by and adapt to the weight status of the social majority at a given school, which may explain in part the lower prevalence of obesity for lower income and African American and Hispanic students who attend economically better off schools in this study. Although evidence is limited on teacher social influence and energy-balance behaviors, longitudinal research on U.S. high school students found that teacher support-in addition to peer and family support-were among the principal factors associated with changes in physical activity [39]. Further research on the various forms of social influences on energy-balance behaviors from peers, parents, teachers and other adult roles models within the school context may provide additional insights into school-level effects on childhood obesity.

At the *school organizational and policy levels*, two studies based on nationally representative data of U.S. secondary school students indicate important SES and ethnic disparities in school-based nutrition and physical activity opportunities. Findings from the 2010 National Secondary School Survey indicate that U.S. middle and high school students attending low SES schools and majority Hispanic and Black schools were less likely to have salads offered at school, less likely to participate in sports programs compared to predominately White or high SES schools, and for students attending low SES schools, less likely to have formal nutrition education or have access to recreational facilities shared outside of school hours [40]. A la carte and vending machines were more prevalent among high SES schools, yet availability of stores or snack bars/carts was significantly higher for middle school Latino students than White or Black students [40]. Although findings from the 2005 School Nutrition and Dietary Assessment study, a nationally representative study of US elementary, middle and high schools, found no ethnic or SES differences in a healthy food environment composite score based on policies and practices, competitive food offerings, and content of USDA lunches, schools with a higher percentage of racial/ethnic minority students were less likely to have a nutrition and health advisory council, and lower income schools were less likely to provide a daily lunch offering of fresh fruit and vegetables [41]. In addition to evidence of SES and ethnic disparities in opportunities for healthy eating and physical activity in U.S. schools, some research indicates lower income and ethnic minority children participate in afterschool program at lower rates [42].

At the *community level*, obesity disparities by school ED may be rooted in the communities in which schools and students reside. Healthier food outlets and opportunities for physical activity in the U.S. have been found to be less available in communities with low SES and high proportions of racial/ethnic minorities [43]. Conversely, U.S. communities that have lower fruit and vegetable prices, higher fast food prices, and greater supermarket availability are associated with higher adolescent fruit and vegetable consumption and lower BMI [44]. In addition to evidence of an inverse association between area-level SES and BMI in middle school girls in Minnesota [45], evidence exists on potential causal factors for children’s obesity via the school setting, such as the association between proximity to fast food restaurants among middle and high schools and lower consumption of fruit and vegetable, higher consumption of soda, and higher obesity in middle and high school students in California [46].

The selected factors described above represent only a small subset of the many environmental forces via the school setting that may influence obesity, and it is likely that these factors do not operate on their own but interact with each other and other individual factors to influence or prevent childhood obesity. In looking forward, research is needed to better understand how factors within family SES, school SES and community SES contexts interact to prevent or promote childhood obesity.

Although students in this study who attended the economically better off schools were generally found to have a lower risk of obesity, this patterning of obesity by school economic disadvantage did not hold for Hispanic children who speak Spanish with their parents, who were found to have high obesity prevalence (~23-24%) across the school economic disadvantage categories. This divergent finding may point to different ‘interactions of influence’ within the school environment that shape the school experience for Spanish-speaking Hispanic children, which may ultimately lessen the potential protective effect of higher income schools on obesity for this subgroup. Lack of English ability has been cited as an important barrier to parents’ access to and comprehension of information on out-of-school programs and education [47], and nationally representative education data from the U.S. indicate that a lower percentage of Spanish-speaking households compared to English-speaking households report receiving personal notes or emails about their child from schools, newsletters, memos or notices [48]. Spanish speaking Hispanic parents have also been found to report lower involvement in schools [49,50], including lower likelihood to report that schools had general meetings, that the school or class held an event that parents could attend, or that the school had opportunities for parents to volunteer [48], as well as greater barriers for involvement such as not feeling welcome, inconvenient meeting times, and language barriers [50]. Some research also indicates cultural differences in body size acceptance, with parents of Hispanic and African American origin more likely to perceive a heavier weight in children as healthy [51,52]. Further research is needed to explore potential differences in obesity-related environments (policy, information, social and built) and cultural factors within lower and higher ED schools as well as how different ethnic and income groups interact with these environments.

### Limitations

This study is based on cross-sectional data, which precludes inferences on the causal relationship between school ED and obesity. While the family SES measure used in this study has some evidence of construct validity based on studies of youth risk behavior [20-22], the measure is based on a single item, which may fail to capture the variability and complexity of parental economic status. This important limitation notwithstanding, several studies have found moderate to good concordance between adolescent and parent report on parent SES [53-56]. It is also worth noting that, while findings generally patterned in a similar fashion for the gender-stratified analyses, fewer findings reached statistical significance as compared to the total sample analyses, which may result from smaller sample size in the stratified analyses. Future research with larger sample sizes may be warranted in investigating gender-specific associations.

## Conclusion

A large body of literature documents the greater health burden and health risks among children from socioeconomically disadvantaged backgrounds [37,57,58] as well as African American and Hispanic children [1,37,59]. Findings from this study provide evidence of an inverse association of childhood obesity with school-level economic disadvantage, underscoring an important need to prioritize economically disadvantaged schools in the fight against childhood obesity. Our findings of lower obesity prevalence among lower income children as well as African American and Hispanic children who attend economically better off schools also provide an important foundation for further research on the role of socioeconomic status at the family, school and community levels for shaping or preventing obesity and obesity-related behaviors in children. The popular axiom “you are a product of your environment” holds specific relevance for understanding how childhood obesity is shaped and prevented, and a school SES contextual lens may provide greater richness to understanding the relationship between the individual and his or her environment, uncover factors that drive disparities in childhood obesity, and better guide school-level interventions aimed at preventing childhood obesity.

## Competing interests

The authors declare that they have no competing interests.

## Authors’ contributions

AS conceived of the study, participated in the design of the original study upon which this paper is based, and drafted the manuscript. LL and NR developed the analytic plan and conducted all analyses, and KM assisted with initial analyses. JD oversaw all data collection and contributed to development of study measures. SK was the Principal Investigator of the original study and assisted with drafting the manuscript. All authors read and approved the final manuscript.

## Declarations

This article has been published as part of *International Journal of Behavioral Nutrition and Physical Activity* Volume 12 Supplement 1, 2015: The science of childhood obesity: an individual to societal framework. The full contents of the supplement are available online at http://www.ijbnpa.org/supplements/12/S1. Publication costs for this supplement were funded by the Michael & Susan Dell Foundation.
